# Dynamics and functional heterogeneity of FOXP3+ regulatory T cells in *Trypanosoma cruzi* infection: a systematic review

**DOI:** 10.1007/s00011-026-02330-6

**Published:** 2026-10-12

**Authors:** Marcelo Eduardo Cardozo, Tatyane Martins Cirilo, Ana Laura Grossi de Oliveira, Ana Rafaela Antunes-Porto, Luisa Vitor do Braga Amaral, José Bryan da Rocha Rihs, Luisa Mourão Dias Magalhães, Lilian Lacerda Bueno, Fabiana Simão Machado, Ricardo Toshio Fujiwara

**Affiliations:** 1 Laboratory of Immunobiology and Parasite Control, Institute of Biological Sciences, Universidade Federal de Minas Gerais, 6627, Antônio Carlos Avenue, Pampulha Campus: Building E4/Room 173, Belo Horizonte, Brazil; 2 Post-graduation Program in Health Sciences: Infectious Diseases and Tropical Medicine, Faculdade de Medicina, Universidade Federal de Minas Gerais, Belo Horizonte, Brazil; 3 Laboratory of Interactions in ImmunoParasitology, Institute of Biological Sciences, Universidade Federal de Minas Gerais, Belo Horizonte, Brazil; 4 Institute of Research in Mucosa and Skin (INCT Mucosa and Skin), Universidade Federal de Minas Gerais, Belo Horizonte, Brazil; 5 Laboratory of Immunoregulation of Infectious Diseases, Institute of Biological Sciences, Universidade Federal de Minas Gerais, 6627, Antônio Carlos Avenue, Pampulha Campus: Building O4/Room 190, Belo Horizonte, Brazil

**Keywords:** Chagas disease, Immunopathology, Immune tolerance, T-cell plasticity, Adaptive immunity, CD39, IL-33, IL-2

## Abstract

**Background:**

The role of regulatory T cells (Tregs) in *Trypanosoma cruzi* infection remains unresolved, with conflicting evidence suggesting either protection from immunopathology or impairment of parasite control.

**Methods:**

We performed the first systematic review integrating data on Treg phenotype and function in human and experimental Chagas disease. A comprehensive search of PubMed, Embase, Scopus, and LILACS (2003–2025) identified studies assessing Tregs during *T. cruzi* infection. Study selection, data extraction, and risk-of-bias assessment followed PRISMA 2020 and Cochrane guidelines.

**Results:**

Twenty-seven studies met eligibility criteria (9 human, 18 murine). Human studies consistently showed that individuals with indeterminate Chagas disease harbor functionally stable Tregs enriched for CD39, CD25^high^, and CTLA-4 expression. In contrast, Chagas cardiomyopathy was associated with Treg instability, PD-1 upregulation, and partial acquisition of Th1-like features. Murine models revealed a phase-dependent role: transient Treg restriction during acute infection enhanced CD8⁺ T cell–mediated parasite control, whereas failure to re-establish regulatory balance promoted chronic cardiac inflammation. Key modulatory pathways included IL-10, IL-2, IL-33/ST2, G-CSF, and CD39.

**Conclusion:**

These findings support a dual-role model in which Tregs dynamically coordinate disease tolerance. Treg-associated signatures, particularly CD39 expression and activated/resting Treg ratios, emerge as promising biomarkers and therapeutic targets for Chagas disease.

**Supplementary Information:**

The online version contains supplementary material available at https://doi.org/10.1007/s00011-026-02330-6.

## Introduction

Chagas disease (CD), caused by the protozoan parasite *Trypanosoma cruzi*, remains a major public health challenge, affecting an estimated 10.5 million people worldwide and representing a leading cause of cardiomyopathy in endemic regions [1]. The clinical course is defined by an acute phase of high parasitemia followed by a chronic phase, which, in 30–40% of individuals, progresses to severe cardiac or digestive complications [2]. This progression is driven by a persistent, dysregulated immune response. Thus, the host inflammatory profile is essential for controlling parasite replication but is also an important driver of the immunopathology that leads to tissue damage [3]. A critical challenge in managing CD is the lack of reliable biomarkers to guide therapy or predict progression. This persistent translational gap continues to limit early risk stratification and the development of target immunomodulatory strategies [4].

The host immune response to *T. cruzi* is dominated by type 1 inflammation, particularly IFN-γ–driven CD8⁺ and CD4⁺ T cell responses that are indispensable for parasite clearance [5]. Recent studies have demonstrated a unique expansion of activated CD4^+^ T cells accompanied by a reduction of functionally competent regulatory T cells, a pattern strongly associated with the onset of Chagas cardiomyopathy [6]. Within this context, regulatory T cells (Tregs), classically defined as CD4⁺CD25⁺FOXP3⁺ lymphocytes, play a pivotal role in maintaining immune homeostasis. Tregs are essential for preventing autoimmunity and controlling excessive inflammatory responses through mechanisms involving cytokines such as IL-10 and TGF-β [7, 8]. The transcription factor FOXP3 acts as the lineage-defining regulator of Treg development and function, and its stable expression is indispensable for suppressive activity [9]. Beyond their canonical regulatory functions, Tregs display substantial phenotypic and functional heterogeneity, expressing molecules such as CD39, ST2, and CTLA-4, and integrating tissue-specific cues that modulate their stability, plasticity, and suppressive potential [10–12].

In the context of *T. cruzi* infection, however, the role of Tregs is highly controversial. Evidence from human chronic disease largely points to Tregs as essential regulators, with their functional capacity correlating with the asymptomatic indeterminate (IND) form [13]. Conversely, some studies have linked dysfunctional Tregs to severe cardiac pathology, implying that excessive or maladapted regulation could hinder parasite clearance and promote tissue damage [14]. In addition, mechanistic studies in experimental murine models often reveal a detrimental role, where Tregs suppress the effector T-cell responses required for pathogen clearance [15]. These divergent observations highlight the possibility that Treg activity is highly context-, time-, and tissue-dependent, shaped by infection stage, antigen load, and microenvironmental cues. Understanding these dynamics is therefore essential to clarify how immunoregulation shapes disease development in CD.

No previous systematic review has comprehensively synthesized and critically appraised the evidence on Treg dynamics across both experimental and human *T. cruzi* infection. Existing studies are fragmented by differences in methodology, infection stage, and phenotypic definitions of Tregs. By systematically addressing these limitations, the present review aims to integrate and evaluate current evidence on Treg frequency, phenotype, and function in CD, consolidating findings from human and animal studies to clarify their complex role and provide a conceptual framework for their application as biomarkers and potential therapeutic targets.

## Methods

### Protocol and registration

This systematic review was conducted in accordance with the Cochrane Handbook for Systematic Reviews of Interventions and reported following the Preferred Reporting Items for Systematic Reviews and Meta-Analysis (PRISMA 2020) statement [16]. The review protocol was registered in the International Prospective Register of Systematic Reviews (PROSPERO) under the reference code #CRD420251158158. The guiding research question was: “What is the role of regulatory T cells as biomarkers or therapeutic interventions in *Trypanosoma cruzi* infection?”.

### Search strategy

A comprehensive literature search was conducted across four electronic databases: PubMed (MEDLINE), Embase, Scopus, and LILACS. The search covered the period from January 2003 to December 2025. The initial search occurred in July 2025, followed by a final updated search in December 2025. The start date of 2003 was justified by the identification of FOXP3 as the lineage-defining marker of regulatory T cells, replacing earlier, non-specific markers such as CD25 alone, which could compromise the accuracy of Treg identification [17].

The search strategy utilized controlled vocabulary from Medical Subject Headings (MeSH), Health Sciences Descriptors (DeCS), and Embase Subject Headings (Emtree). Terms related to the condition and the cell type were combined using Boolean operators (AND, OR), including: “Chagas disease”, “*Trypanosoma cruzi*”, “T-Lymphocytes, Regulatory”, “Treg”, and “Regulatory T cells”. No restrictions regarding language or document type were applied during the search phase. Complete search strings for all databases are provided in Supplementary Table 1. Reference lists of all included studies and relevant reviews were also manually screened to identify additional eligible articles.

### Eligibility criteria

This review included both human observational or interventional studies and in vivo preclinical studies that evaluated the role, frequency, phenotype, or function of Tregs during *T. cruzi* infection.

Studies were excluded if they met any of the following criteria: [1] exclusively ex vivo or in vitro studies without in vivo infection context; [2] publication types such as case reports, protocols, letters to the editor, editorials, reviews, and conference abstracts; [3] studies published before 2003; [4] studies that did not utilize FOXP3 as a definitive marker for Treg characterization; [5] articles lacking original data on Treg frequency, absolute number, phenotype, or function; [6] studies mentioning Tregs solely in the introduction or discussion without presenting specific results; [7] studies evaluating interventions with indirect effects on Tregs without direct measurement of this cell population; and [8] studies with insufficient clinical characteristics or missing data essential for extraction. No language or publication-status restrictions were applied. Reference lists of all included articles and relevant reviews were also screened manually to identify additional eligible studies.

### Study selection and data extraction

References retrieved from the databases were imported into the Rayyan QCRI software [18] for duplicate removal and screening. The selection process occurred in two stages. First, two independent reviewers (MEC and ARAP) screened titles and abstracts against the eligibility criteria. In the second stage, the full texts of potentially eligible studies were retrieved and assessed independently by the same reviewers.

Data extraction was performed using a standardized form. The extraction form was pilot-tested on a sample of studies to ensure consistency. The following variables were extracted: first author, year of publication, country, study aim, study design, Treg gating/identification strategy, population characteristics (sample size, sex, age, clinical form/animal strain), Treg-related outcomes (frequency, phenotype, functionality, mechanistic pathways), and key findings regarding the role of Tregs in *T. cruzi* infection. Disagreements at any stage were resolved through consensus or consultation with a third reviewer (ALGO).

### Risk of bias assessment and data analysis

Two reviewers (JBRR and MEC) independently assessed the methodological quality and risk of bias of the included studies. Discrepancies were resolved by consensus or arbitration by a third reviewer (ALGO).

Animal Studies: For preclinical studies, the risk of bias was assessed using the Collaborative Approach to Meta-Analysis and Review of Animal Experimental Studies (CAMARADES) checklist. Studies were evaluated on 10 domains, including peer-reviewed publication, random allocation, blinded outcome assessment, sample size calculation, and compliance with animal welfare regulations.

Human Studies: The assessment tools were selected based on the specific study design. Randomized Controlled Trials: The Cochrane Risk of Bias 2 (RoB 2) tool was used to assess domains such as randomization process, deviations from intended interventions, and missing outcome data. Non-Randomized Interventional Studies: The Risk Of Bias In Non-Randomized Studies—of Interventions (ROBINS-I) tool was employed to evaluate confounding, selection of participants, and measurement of outcomes. Observational Studies: For cross-sectional studies, the Joanna Briggs Institute (JBI) Critical Appraisal Checklist was used. This tool assesses methodological rigor through questions regarding comparability of groups, reliable measurement of exposure and outcomes, and appropriate statistical analysis, with items scored as “Yes”, “No”, “Unclear”, or “Not Applicable”.

Data management and initial curation were performed using Microsoft^®^ Excel. Data manipulation and graphical rendering were executed using RStudio/Posit version 2025.05.0, Build 496, and the following packages: dplyr (v1.1.4), ggplot2 (v3.5.2), and ggalluvial (v0.12.5).

Given the methodological heterogeneity across the included studies, a formal statistical meta-analysis was not feasible. Data synthesis was therefore conducted in three sequential and complementary steps, each designed to address a distinct analytical question. Mechanistic categorization (qualitative subgroup analysis): to identify recurring mechanistic patterns across studies, two independent reviewers (MEC and TMC) systematically coded each included study according to the primary Treg-related biological pathway reported. Mechanistic categories were defined a priori and comprised: (i) suppressive pathways (e.g., CD39-mediated purinergic signaling, CTLA-4 co-inhibition, IL-10 production); (ii) survival and lineage-maintenance pathways (e.g., IL-2, G-CSF); (iii) tissue-repair programs (e.g., IL-33/ST2 axis, amphiregulin production); and (iv) dysfunctional or unstable phenotypes (e.g., PD-1-associated exhaustion, Th1-like reprogramming).

To visualize the relationships between mechanistic themes, study model, infection phase, and functional outcome, an alluvial diagram was constructed. In this diagram, each stratum represents a categorical variable (model → phase → mechanism → outcome), and each flow stream represents one or more studies sharing the same mechanistic association along that axis. This visualization was designed to map co-occurrence patterns and directionality among mechanistic variables across the evidence base, not to quantify effect sizes, and must be interpreted as an evidence-mapping tool.

Finally, to complement the mechanistic visualization with a directional quantitative synthesis, a vote counting analysis based on direction of effect was performed, as recommended by the Cochrane Handbook for systematic reviews in which heterogeneity precludes formal pooling. Each included study was classified according to the direction of the reported Treg effect: protective, detrimental, context-dependent, or not applicable (N/A). Counts were then stratified by study model (human vs. murine) and infection phase (acute, chronic, or both). To formally test whether the proportion of studies reporting a protective Treg role differed from chance, a one-sample binomial test was applied, using 0.5 as the null probability (i.e., equal likelihood of protective vs. detrimental effect). Studies classified as context-dependent or N/A were excluded from the binomial test, as their directional assignment was indeterminate.

## Results

**Fig. 1:**
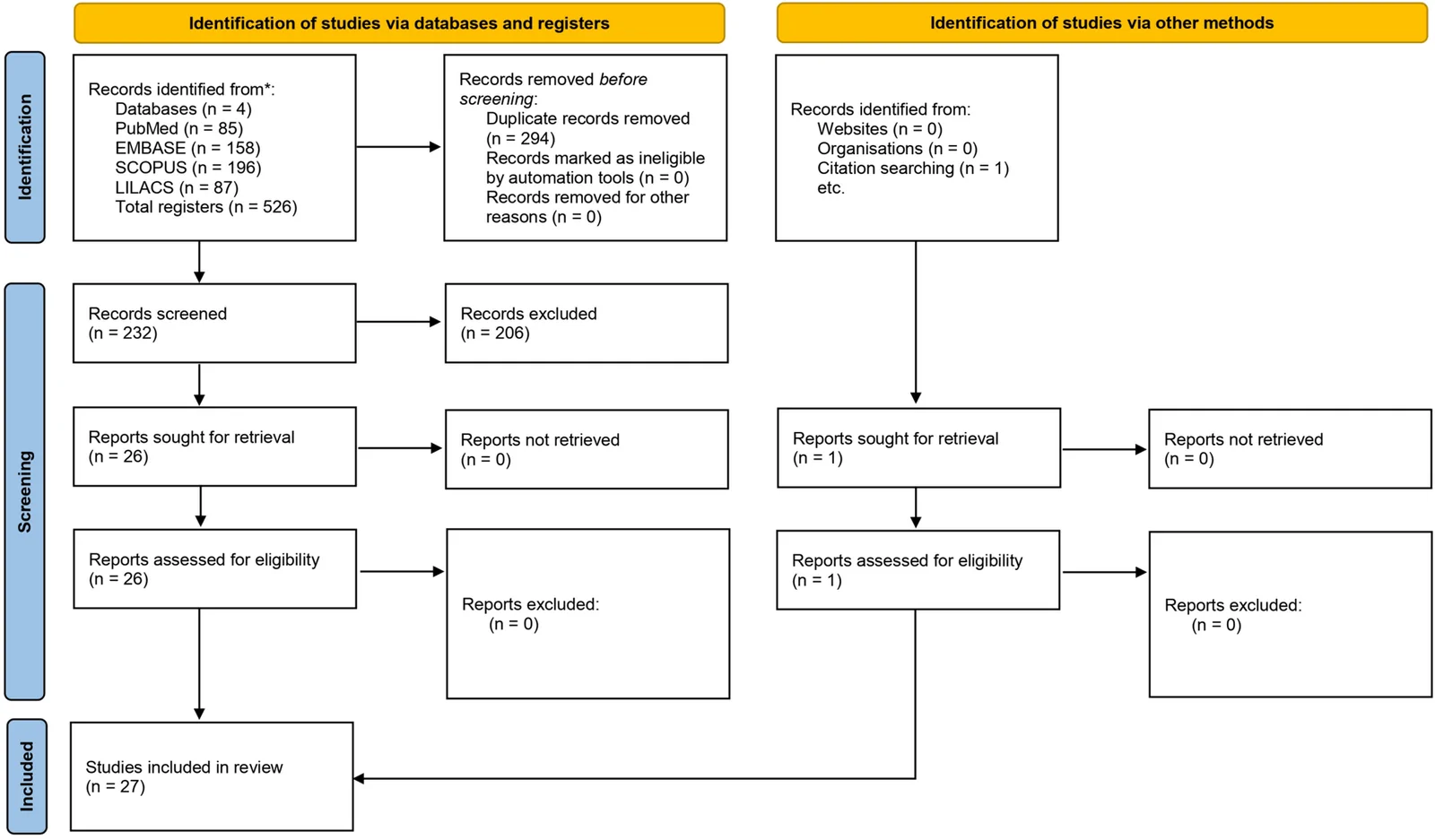
PRISMA flow chart of the study selection and inclusion process

The systematic search identified a total of 526 records across the four electronic databases (PubMed, Embase, Scopus, and LILACS). After removing 294 duplicates, 232 unique records were screened by title and abstract. Of these, 206 were excluded because they did not meet the predefined eligibility criteria. All 26 articles assessed in full text were included in the review. Additionally, one study identified through citation searching met the criteria, resulting in a total of 27 included studies. The selection process is detailed in Fig. 1 (PRISMA flow diagram).

The included studies comprised 9 (33.3%) human studies (observational and interventional; all focusing on chronic Chagas disease) [6, 13, 14, 19–24], and 18 (66.7%) animal studies [15, 25–41] performed in mouse models (A/J, BALB/c, C57BL/6) infected with diverse *T. cruzi* strains. All included studies used FOXP3 as a definitive marker for Tregs and provided original immunological data on Treg frequency, phenotype, function, or mechanistic contribution during *T. cruzi* infection.

Human studies were conducted in Brazil (8/9) and Spain (1/9), published in journals with impact factors ranging from 1.6 to 8.4. Participants were adults across a broad age range, typically 18 to 75 years, with most studies reporting a balanced sex distribution or a slight predominance of females (approximately 45–57%, when reported). Sample sizes varied substantially (from 14 to 202 participants), and all studies compared individuals with indeterminate (asymptomatic) and/or cardiac forms of chronic CD, often including non-infected controls. One randomized clinical trial, Vallejo et al. [23] (1, 11.1%) investigated the effect of pharmacological treatment. One non-randomized interventional study, Llaguno et al. [21] (1, 11.1%), examined post-treatment immune alterations, and the remaining studies (7, 77.8%) had observational design. All human studies consistently defined Tregs using FOXP3 expression, typically through CD4⁺CD25^high^FOXP3⁺ gating on PBMCs or whole blood, with some studies incorporating antigen-stimulated assays.

Animal studies were predominantly conducted in Argentina and Brazil, published in journals with impact factors ranging from 1.8 to 7.6. Experiments were performed in mice (A/J, BALB/c, and C57BL/6 mice; both sexes; 4–12 weeks old), infected with a variety of *T. cruzi* strains: Y (6, 33%), Tulahuén (10, 55.6%), Brazil (2, 11.1%), and Colombiana (1, 5.6%) (some studies employed multiple strains). Studies primarily evaluated the acute phase of infection [26, 30–32, 34, 36, 37, 40] or both the acute and chronic phases [15, 25, 27–29, 33, 35, 39, 41]. Notably, one study by Vasconcelos et al. [38] focused exclusively on the chronic phase. It should be noted that the classification of acute and chronic phases in murine *T. cruzi* infection is not universally standardized and varies considerably depending on the parasite strain, mouse genetic background, inoculum size, and infection route. The temporal framework adopted in this review should therefore be understood as a conceptual approximation.

Furthermore, experimental studies frequently utilized genetically modified models to assess the role of various immune mediators in T regulatory cell (Treg) dynamics. These models included: DEREG (Treg knockout mouse) [15]; ST2 KO [25]; CD39 KO [15]; FPR2 KO [28]; AhRd (low-affinity Aryl hydrocarbon receptor) [27]; B-cell KO [31]; and Ebi3 KO [32]. Tissue sources varied and included spleen, liver, heart, and lymph nodes, providing complementary insights into systemic and tissue-specific regulation. Sample sizes in animal studies varied widely, ranging from 3 to 25 animals per group, depending on the experimental model and study design. A detailed summary of study characteristics is provided in Table 1.

**Table 1:** Main characteristics of the included studies in the systematic review

Article	Country	Journal/ impact factor	Study design/phase of infection	Patient cohort/model characteristics	Population characteristics	Sample (*n*)	Treg definition (gating) and source
*Human studies*							
Medeiros et al. [19]	Brazil	Microbes & Infection Impact Factor: 2.7	Cross-sectional	IND versus CARD versus NI	Age: 18–69 Females: 50–56% (across groups)	*N* = 202 (70 NI, 71 CARD, 61 IND)	CD4^+^CD25^high^FOXP3^+^ PBMC
Almeida et al. [6]	Brazil	Journal of Translational Medicine Impact Factor: 8.4	Cross-sectional	IND vs. Mild/Severe CARD vs. NI	Age: 18–65 Females: n/a	*N* = 38 (14 NI, 8 IND, 9 Mild, 7 CARD)	CD4^+^CD25^+^ FOXP3^+^ PBMC
Pinto et al. [20]	Brazil	Frontiers in Cardiovascular Medicine Impact Factor: 2.9	Cross-sectional (In vitro CD80/CD86 blockade)	IND vs. CARD vs. NI	Age: 30–75 Females: n/a	*N* = 29 (9 NI, 9 IND, 11 CARD)	CD4^+^CD25^high^FOXP3^+^ PBMC (Ag- stimulated)
Llaguno et al. [21]	Brazil	Infection & Immunity Impact Factor: 2.9	Longitudinal (48mo follow-up post-BZN)	IND vs. CARD	Age: 18–65 (IND) Females: 50% (IND), 44% (CARD)	*N* = 17 (8 IND, 9 CARD)	CD4^+^CD25^high, neg or low^ FOXP3^+^ PBMC
Damasio et al. [22]	Brazil	Human Immunology Impact Factor: 2.2	Cross-sectional	IND vs. CARD vs. NI	Age: 29–70 Females: n/a	*N* = 30 (10/group)	CD4^+^CD25^high^FOXP3^+^ Whole blood
Almeida et al. [14]	Brazil	Scandinavian Journal of Immunology Impact Factor: 1.6	Cross-sectional	IND vs. Mild/Severe CARD	Mean age: n/a Females: 45–65% (across groups)	*N* = 48 (17 IND, 31 CARD)	CD4^+^CD25^+^FOXP3^+^ Whole blood (Ag- stimulated)
Vallejo et al. [23]	Spain	Acta Tropica Impact Factor: 2.5	Randomized clinical trial	IND (BZN-treated vs. Untreated)	Mean age: 35 Females: 57%	*N* = 14 (7 treated, 7 non-treated)	Tregs subsets defined as: **Suppressor Treg**: FOXP3^high^ **Resting Treg**: FOXP3^low^CD45RA^+^ CD127^Low^ **Activated Treg**: CD25^+^FOXP3^high^ CD45RA^−^CD127^low^. **Non-suppressor Treg**: CD25^+^FOXP3^low^ CD45RA^−^CD127^low^. Whole blood
Araújo et al. [24]	Brazil	Immunobiology Impact Factor: 2.3	Cross-sectional (In vitro suppression assay)	IND vs. CARD vs. NI	Age: 25–70 Females: n/a	*N* = 48 (11 NI, 22 IND, 15 CARD).	CD4^+^CD25^high^FOXP3^+^ Whole blood
Guedes et al. [13]	Brazil	PLoS Neglected Tropical Disease Impact Factor: 3.4	Cross-sectional (BZN-treated vs. Untreated)	IND vs. CARD vs. NI	Age: 42–63 Females: 48%	*N* = 39 (18 IND, 11 CARD, 10 NI)	CD4^+^CD25^high^FOXP3^+^ PBMC
*Animal models (Murine)*							
Cardozo et al. [25]	Brazil	ACS Infec Diseases. Impact Factor: 3.8	Acute & Chronic WT vs. ST2 KO	Y Strain (DTU TcII) (10^3^ tryps)	BALB/c (F), 8–10 wk	*N* = 4–8/group	CD4^+^FOXP3^+^ Heart
Boccardo et al. [26]	Argentina	Plos Pathog Impact Factor: 4.9	Acute infection IL-33/IL-2 Treatment	Tulahuén (DTU TcVI) (5 × 10^3^-10^4^ tryps)	C57BL/6 (M/F), 8–12 wk	*N* = 3–10/group	trTregs: CD4⁺FOXP3⁺ST2⁺ KLRG1⁺ Spleen, liver and skeletal muscle
Araujo Furlan et al. [15]	Argentina	Plos Pathog Impact Factor: 4.9	Acute & Chronic Treg depletion / Adoptive transfer	Tulahuén (DTU TcVI) (5 × 10^3^ tryps)	C57BL/6 (M/F), 8–12 wk DEREG, CD39 KO	*N* = 3–10/group	FOXP3⁺ CD4⁺CD39⁺ Blood, spleen, liver
Ambrosio et al. [27]	Argentina	Frontiers in Immunology Impact Factor: 5.9	Kinetics of Tregs (Acute & Chronic)	Tulahuén (DTU TcVI) (5 × 10^4^ tryps)	BALB/c vs. C57BL/6 (M/F) 6–8 wk	*N* = 3/time point	CD4^+^CD25^+^FOXP3^+^ Spleen
Pereira et al. [28]	Brazil	Cellular Immunology Impact Factor: 2.9	Acute & Chronic WT vs. FPR2 KO	Y Strain (DTU Tc II) (10^3^ tryps)	C57BL/6 (F), 8–10 wk	*N* = 3–9/group	CD4^+^CD25^+^FOXP3^+^ Spleen
Ambrosio et al. [29]	Argentina	Frontiers in Immunology Impact Factor: 5.9	Acute & Chronic AhR modulation	Tulahuén (DTU Tc VI) (50 × 10^4^ tryps)	BALB/c, C57BL/6, AhRd 6–8 wk	*N* = 3–5 /group	CD4^+^CD25^+^FOXP3^+^ Spleen, heart and liver
Araujo Furlan et al. [30]	Argentina	Frontiers in Immunology Impact Factor: 5.9	Functional analysis of Treg Acute phase	Tulahuén (DTU Tc VI) (5 × 10^3^ − 5 × 10^4^ tryps)	C57BL/6 (FOXP3-GFP) 7–10 wk	*N* = 3–11 /group	CD4^+^CD25^+^FOXP3^+^ Spleen, liver and lymph nodes
Gorosito Serran et al. [31]	Argentina	Frontiers in Immunology Impact Factor: 5.9	Acute phase WT vs. B-cell KO (muMT)	Brazil Strain (DTU Tc II) (10^4^ tryps)	C57BL/6 vs. muMT 7–9 wk	*N* = 4–8/group	CD3^+^CD4^+^FOXP3^+^ Spleen, Liver, and Lymph Nodes
Medina et al. [32]	Brazil	Frontiers in Immunology Impact Factor: 5.9	Acute phase WT vs. Ebi3 KO	Y Strain (DTU Tc II) (10^3^ tryps)	C57BL/6 (F), 6–8 wk	*N* = 5–8/group	CD4^+^ FOXP3^+^; (Focus on Tr1 cells: CD4⁺FOXP3⁻IL-10⁺) Spleen
Prochetto et al. [33]	Argentina	Oncotarget Impact Factor: -	Acute & Chronic Immunization (TSf + ISPA)	Tulahuén (DTU Tc VI) (10^3^ tryps)	BALB/c (F), 6–8 wk	*N* = 4–10/group.	CD4⁺FOXP3⁺ Spleen
Ersching et al. [34]	Brazil	PLoS Pathog Impact Factor: 4.9	Acute *T. cruzi*-exposed DC / Treg depletion	Y strain (DTU Tc II) (10^4^ tryps)	C57BL/6 (F), 8–12 wk DEREG	*N* = 4/group	CD4⁺FOXP3⁺ Spleen
González et al. [35]	Argentina	PLoS Negl Trop Dis Impact Factor: 3.4	Acute and chronic Thymic tTregs	Tulahuén & Y (DTUs Tc VI and Tc II) (10^2^ − 10^3^ tryps)	BALB/c vs. C57BL/6 (M), 6–8 wk	*N* = 3–6/group	Thymus-derived Tregs (tTregs): FOXP3⁺ CD4+
González et al. [36]	Argentina	Brain, Behavior & Immunity Impact Factor: 7.6	Acute IL-2 + Dexamethasone	Tulahuén (DTU Tc VI) (10^3^ tryps)	C57BL/6 (M), 8–10 wk	*N* = 4–10/group	CD4^+^FOXP3^+^ Spleen
Bonney et al. [37]	USA	Parasitology Research Impact Factor: 1.8	Acute Anti-CD25 depletion	Brazil Strain (DTU Tc II) (10^4^ tryps)	A/J (M), 4–6 wk	*N* = 3–5/group	CD4^+^CD25^+^FOXP3^+^ Spleen
Vasconcelos et al. [38]	Brazil	FASEB J Impact Factor: 4.2	Chronic G-CSF treatment	Colombian (DTU Tc I) (100 tryps)	C57BL/6 (M), 4 wk	*N* = 9–10/group	CD3^+^CD4^+^CD25^+^ FOXP3^+^ Heart and spleen
Sanoja et al. [39]	Spain/ Venezuela	Plos One Impact Factor: 2.9	Acute & Chronic C57BL/6 vs. BALB/c	Y (DTU Tc II) (50–2 × 10^3^ tryps)	BALB/c and C57BL/6 Female mice, 6–8 wk	*N* = 25–25/group.	CD4^+^CD25^+^ FOXP3 Heart
Flores- García et al. [40]	Mexico	BioMed Res Int Impact Factor: 2.3	Acute rSSP4 Immunization	Tulahuén (DTU Tc VI) (10^3^ tryps)	BALB/c (F), 5–10 wk	*N* = 3–4/group	CD4⁺CD25^+^FOXP3⁺ Spleen
Knubel et al. [41]	Argentina	Plos One Impact Factor: 2.9	Acute & Chronic 3- Hydroxykynurenine treatment	Tulahuén (DTU Tc VI) (500 tryps)	BALB/c (F), 6–8 wk	*N* = 4–8/ group	CD4^+^CD25^+^FOXP3^+^ Spleen

Ag, Antigen; AhR, Aryl hydrocarbon Receptor; BM-DC, Bone marrow-derived dendritic cells; BZN, Benznidazole; CARD, Patients with Chronic Chagas Cardiomyopathy; DEREG, Depletion of Regulatory T cells (transgenic mouse model); dpi, Days post-infection; DTU, discrete typing units; F, Female; G-CSF, Granulocyte-colony stimulating factor; IF, Impact Factor; IND, Indeterminate form; KO, Knockout; M, Male; NI, Non-Infected controls; PBMC, Peripheral Blood Mononuclear Cells; Treg, Regulatory T cell; tryps, Trypomastigotes; WT, Wild-type

As summarized in Table 2, the evidence gathered across 27 studies reveals convergent yet stage-dependent patterns of Treg dynamics during *T. cruzi* infection. Among human investigations (*n* = 9), most studies (7, 77.8%) identified Tregs as predominantly protective, particularly in individuals with the indeterminate clinical form, where Tregs displayed a stable suppressive phenotype marked by high FOXP3, CD25, and notably CD39 expression. Only one study (11.1%) associated Tregs with detrimental regulation through PD-1 overexpression and bias towards the Th1 profile, while another (11.1%) reported context-dependent effects, with the Treg phenotype changing according to the state of immune activation due to parasitological treatment.

**Table 2:** Dynamics of Regulatory T cells and infection outcomes in humans and mice

Article	Comparative events	Treg dynamics & phenotype	Clinical/pathological outcome	Role
*Human studies*				
Medeiros et al. [19]	FOXP3 polymorphisms	FOXP3 promoter polymorphism (-3499 G/T) is prevalent in IND females	Polymorphism associated with higher Treg frequency and maintenance of IND form	Protective
Almeida et al. [6]	Cardiomyopathy development	Mild CARD: ↓ CD39 subsets Severe CARD: ↑ Cytotoxic CD4^+^ (GrzB ^+^ Perforin^+^)	Loss of functional CD39^+^ Tregs marks the onset of cardiac damage	Protective
Pinto et al. [20]	CD80/CD86 blockade	IND: Activation relies on CD86–CD86-CTLA-4 axis CARD: Blockade of CD80 increases CD8^+^ Tregs	Differential engagement of co-stimulatory pathways (CD80 vs. CD86) dictates regulatory control failure in CARD	Protective
Llaguno et al. [21]	Post-BZN Follow-up	IND: ↑ CD25^high^ Tregs CARD: ↑ CD25^low^ Tregs	Preservation of CD25 Tregs and balanced cytokines (IFN-γ/IL-10) associates with non-progression	Protective
Damasio et al. [22]	Immune profile	IND: ↑ CD39 expression CARD: ↑ PD-1 expression	CD39 is linked to effective suppression; PD-1/PD-L1 axis is linked to cellular dysfunction and cardiomyopathy	Protective
Almeida et al. [14]	Immune profile	CARD: ↑ CD25^high^ in severe disease	High Treg frequency correlates with worse LVEF, suggesting compensatory expansion or functional failure	Detrimental
Vallejo et al. [23]	BZN-treated vs. untreated	Treated: ↓ Total Tregs; shift to Resting phenotype Untreated: ↑ Activated Tregs responding to inflammation	Treg reduction in treated patients tracked with decreased immune activation. Highly activated Tregs in untreated patients are linked to inflammation	Context-dependent
Araújo et al. [24]	Immune profile	IND: Suppressive profile (IL-10^+^; IL-17^+^). CARD: Inflammatory profile (IL-6; IFN-γ; TNF)	Higher Treg frequency in IND correlates with better cardiac function (High LVEF, Low LVDD)	Protective
Guedes et al. [13]	Immune profile	Similar Treg frequency across groups IND: Higher suppressive function of Tregs (CTLA-4, FOXP3^high^)	Preserved Treg suppressive activity correlates with higher LVEF	Protective
*Animal models (Murine)*				
Cardozo et al. [25]	ST2 KO	Heart Treg numbers are unchanged, but ST2 signaling leads to a higher inflammatory profile	Lack of ST2 axis leads to exacerbated IFN-γ myocarditis and heart dysfunction	N/A
Boccardo et al. [26]	IL-33/IL-2 treatment	Infection depletes trTregs. IL-33/IL-2 treatment restores them	Restoration of trTregs reduces tissue damage and improves parasite control.	Protective
Araujo Furlan et al. [15]	CD39 KO/depletion	CD39 expression on Tregs is required for CD8^+^ T cell suppression	Treg depletion (or CD39 removal) allows a robust CD8^+^ response and better parasite clearance	Detrimental
Ambrosio et al. [27]	IDO/AhR Axis (B6 vs. BALB)	B6 mice fail to expand Tregs due to impaired IDO/Kynurenine pathway.	Failure to induce Tregs leads to exacerbated inflammation in B6 mice.	Protective
Pereira et al. [28]	FPR2 KO	Acute: ↑ Treg proportion. Chronic: ↓ Treg proportion	Acute Treg expansion aids parasite control; chronic loss leads to myocarditis.	Context-dependent
Ambrosio et al. [29]	AhR modulation	Agonist: ↑ Tregs (TGF-β, IL-10). AhRd (Low affinity): ↓ Tregs	Agonist: ↑ Parasite load. AhRd: Stronger Th1 response and better parasite control	Detrimental
Araujo Furlan et al. [30]	Adoptive transfer of tregs	Natural infection: ↓ Treg frequency Transfer: Restores numbers but limits CD8 + response	Early increase in Tregs suppresses protective CD8^+^ immunity, worsening parasite control	Detrimental
Gorosito Serran et al. [31]	B-cell deficient (muMT)	↓ Treg (IL-10^+^CD39^+^) in absence of B-cells	Reduced Tregs correlates with uncontrolled effector response, inflammation, and mortality	Protective
Medina et al. [32]	Ebi3 (IL-27) KO	Focus on Tr1 (IL-10^+^ FOXP3^−^) rather than classical Tregs	Ebi3/IL-27 axis induces protective Tr1 response; deficiency causes fatal myocarditis	Protective
Prochetto et al. [33]	TSf-ISPA vaccine	Immunization increases splenic CD4 + FOXP3^+^ cells	Effective vaccine balances effector immunity (parasite control) with Tregs (tissue protection)	Protective
Ersching et al. [34]	DC-Treg interaction	*T. cruzi*-exposed DCs induce suppressive Tregs (CTLA-4/TGF-β dependent)	Tregs suppress protective CD8^+^ priming. Depletion transiently improves control	Detrimental
González et al. [35]	Thymic atrophy	Severe loss of thymic Tregs (tTreg) during acute infection	Loss of the tTreg pool contributes to long-term immune dysregulation	Protective
González et al. [36]	IL-2 + dexamethasone treatment	Natural Tregs acquire Th1-like (T-bet^+^) features. IL-2 + Dex treatment restores proliferation and profile	Boosting Tregs (IL-2 + Dex) improves survival, counteracting the natural limit on Treg response.	Protective
Bonney et al. [37]	Anti-CD25 depletion	Depletion reduces Tregs (functional ablation)	Depletion leads to higher Th1/Th17 responses. Enhanced parasite clearance and reduced myocarditis	Detrimental
Vasconcelos et al. [38]	G-CSF treatment	Treatment increases Tregs in heart/spleen, and promotes a shift to IL-10/TGF-β profile	Reduced fibrosis, myocarditis, and parasitism	Protective
Sanoja et al. [39]	Resistant (B6) vs. Susceptible (BALB)	B6: ↑ Treg cardiac infiltration; high FOXP3/FR4. BALB/c: ↓ cardiac Tregs.	Co-existence of Th1 response + Tregs in B6 heart permits parasite clearance without immunopathology	Protective
Flores-García et al. [40]	rSSP4 immunization	Induction of Antigen-specific Tregs (TGF-β mediated).	Increased parasitemia but lower cardiac inflammation and mortality	Context-dependent
Knubel et al. [41]	3-HK treatment	3-HK promotes Treg development and TGF-β response	Amelioration of chronic cardiac lesions and ECG abnormalities	Protective

↑, increase; ↓, decrease; 3-HK, 3-Hydroxykynurenine; Ag, Antigen; AhR, Aryl hydrocarbon Receptor; BZN, Benznidazole; CARD, Patients with Chronic Chagas Cardiomyopathy; DC, Dendritic Cell; G-CSF, Granulocyte-colony stimulating factor; IND, Indeterminate form; KO, Knockout; LVDD, Left Ventricular Diastolic Dysfunction; LVEF, Left Ventricular Ejection Fraction; N/A, Not applicable; NI, Non-Infected; ST2, Interleukin 33 receptor; tTreg, Thymic Regulatory T cell; trTreg, Tissue-resident Regulatory T cell; Tr1, Type 1 Regulatory T cell

**Fig. 2:**
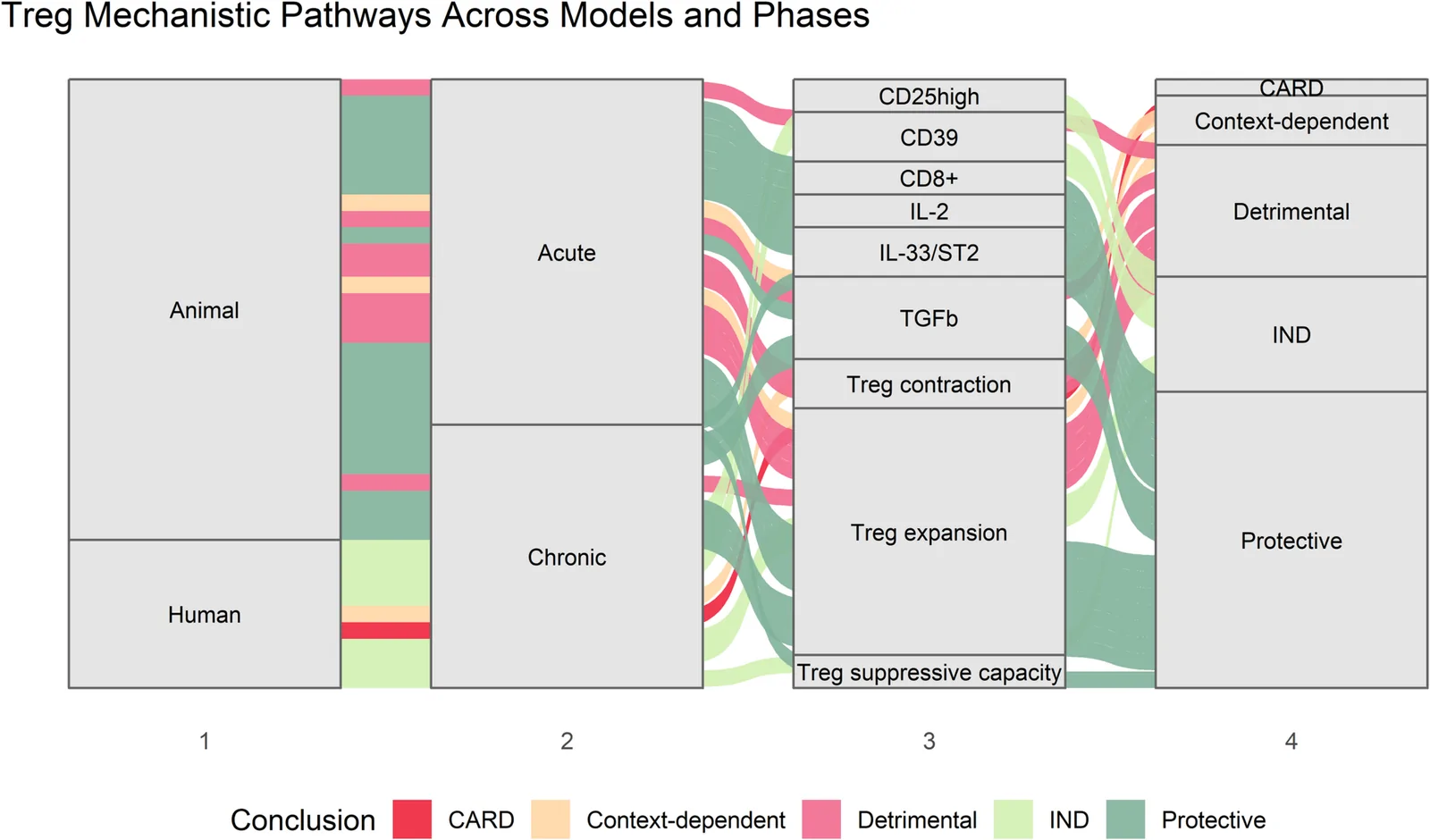
Mechanistic pathways of Treg function across experimental models and infection phases in *T. cruzi*infection. Alluvial plot depicting the flow of mechanistic themes from [1] study model (animal or human) [2], infection phase (acute or chronic) [3], specific Treg mechanisms, and [4] study conclusions (protective, detrimental, cardiac involvement, indeterminate, or context-dependent). Each stream represents one or more studies reporting the same mechanistic association. Only mechanistic elements that appeared significantly and reproducibly in more than one study were included, ensuring that the visualization represents robust and cross-validated findings. Colors correspond to the final interpretation of Treg function (protective, detrimental, cardiac involvement, indeterminate, or context-dependent)

**Fig. 3:**
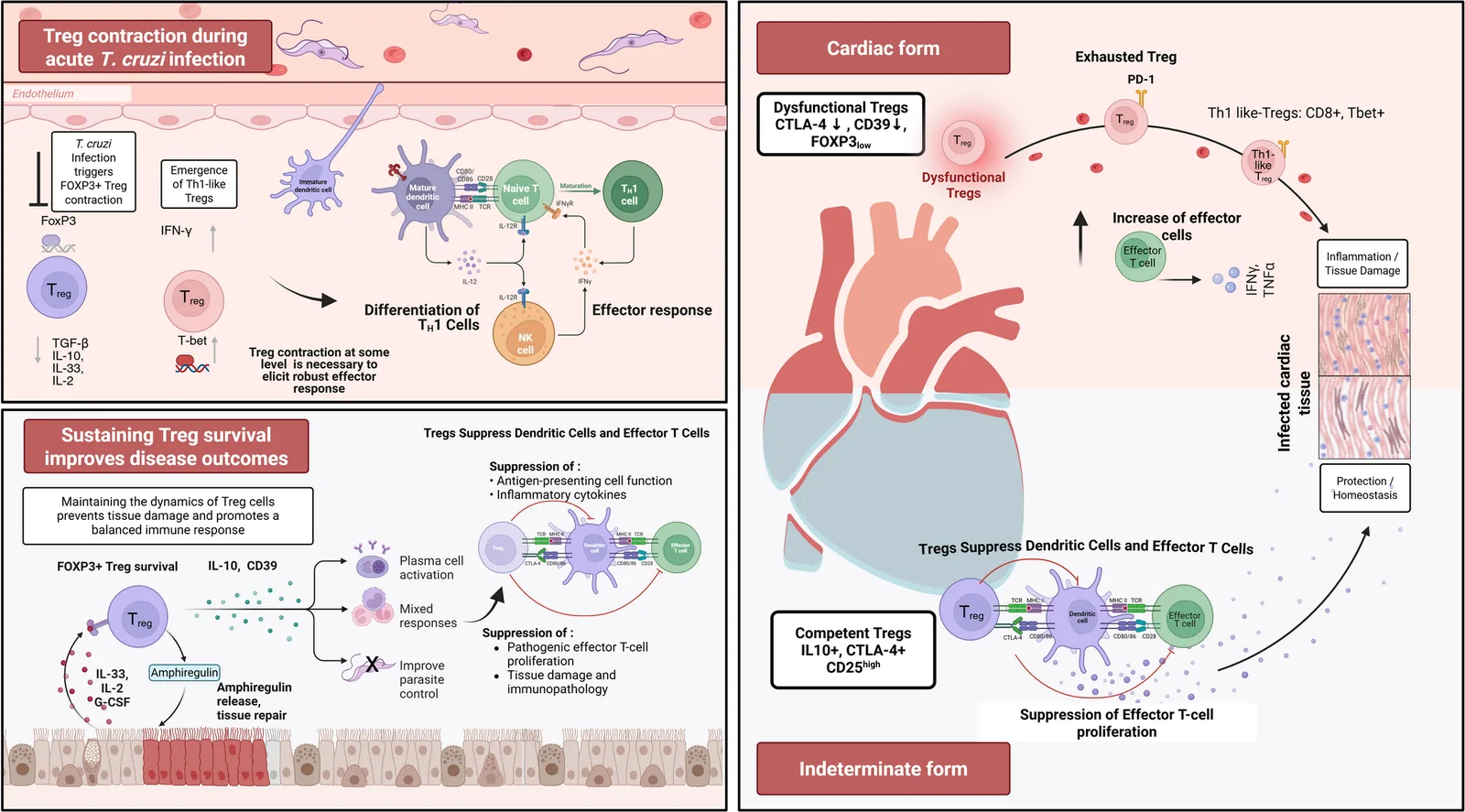
Regulatory T-cell (Treg) Dynamics in *Trypanosoma cruzi* Infection. During acute infection, a transient Treg contraction facilitates robust Th1 effector responses essential for parasite control. Crucially, the outcome of chronic Chagas disease depends on Treg function. In the severe Cardiac form, Tregs are exhausted (PD-1 upregulated) and exhibit impaired suppression, leading to uncontrolled inflammation and heart damage. Conversely, the benign Indeterminate form is sustained by functionally competent Tregs (CTLA-4⁺, IL-10⁺) that preserve immune homeostasis and protect the heart from inflammatory injury. Figure created on Biorender.com

Animal studies (*n* = 18) showed greater heterogeneity, with over half of them, 10 (55.6%) were reported to have protective regulatory activity, whereas 5 (27.8%) demonstrated detrimental Treg effects, primarily when regulatory activity suppressed early effector responses required for parasite control. Two studies (11.1%) described phase-specific or mouse background-dependent effects, which suggest that tissue microenvironments and divergent parasite–host interactions influence the stability of the regulatory compartment. Across species, recurrent mechanistic themes were identified (the main mechanistic pathways are highlighted in Fig. 2). In humans, CD39 consistently emerges as a hallmark of regulatory stability and favorable immune modulation, positioning this ectonucleotidase as a compelling biomarker candidate for disease progression and therapeutic monitoring. Conversely, heightened PD-1 expression, frequently accompanied by partial effector-like reprogramming of Tregs, is linked to regulatory dysfunction and the development of chronic cardiomyopathy. Genetic determinants further modulate these outcomes: the FOXP3 − 3499 G/T polymorphism, for example, was uniquely associated with increased susceptibility among women, suggesting sex-specific regulatory pathways. Experimental studies manipulating IL-2, IL-33, G-CSF, AhR/IDO signaling, or Treg depletion, demonstrated that the immunological impact of Treg modulation is temporal: early attenuation of Tregs is advantageous for parasite control, whereas restoration of regulatory tone is indispensable for limiting immunopathology (Fig. 3).

**Fig. 4:**
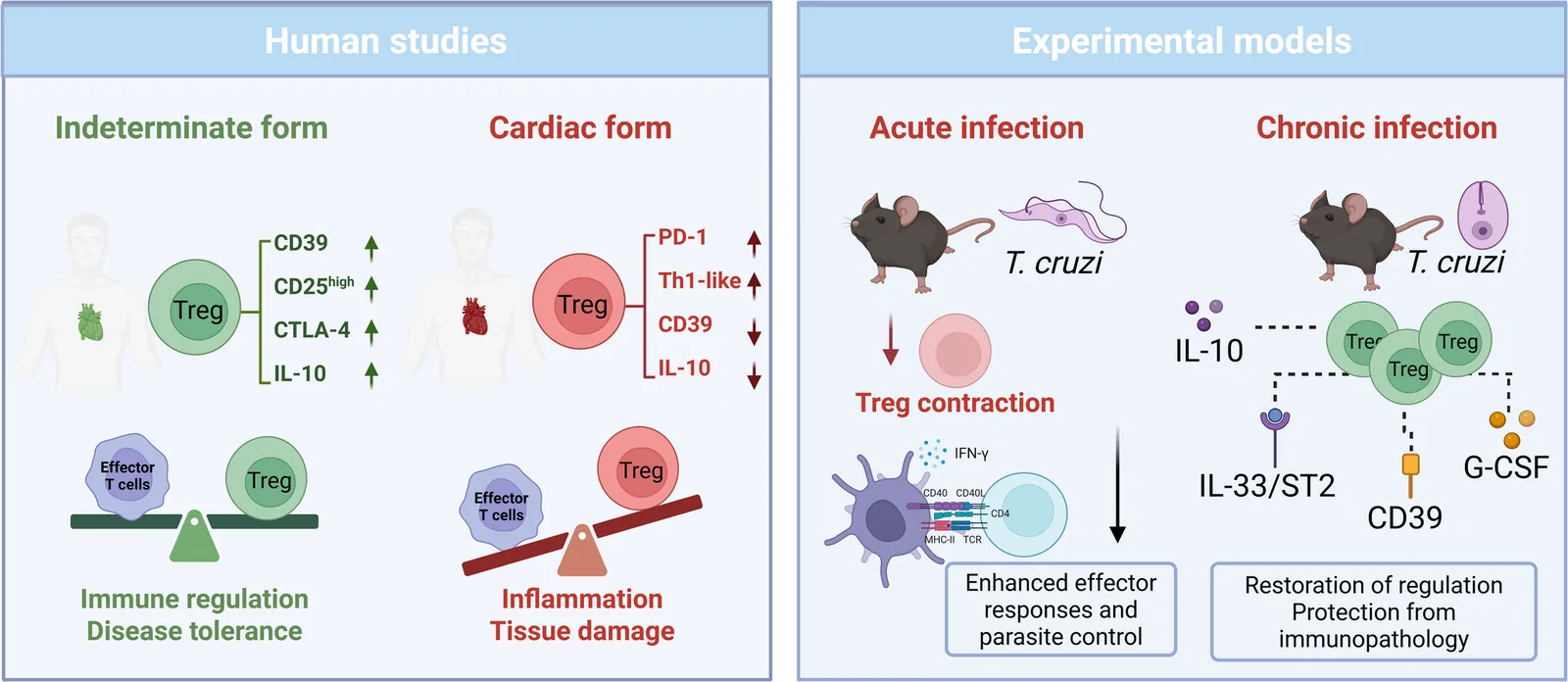
Integrative framework linking human and experimental evidence on regulatory T-cell biology in Chagas disease. Human studies consistently indicate that the indeterminate clinical form is associated with stable and functionally competent FOXP3 + regulatory T cells (Tregs), characterized by increased expression of CD39, CD25^high^, CTLA-4, and IL-10, supporting immune regulation and disease tolerance. In contrast, patients with Chagas cardiomyopathy exhibit dysfunctional Tregs with increased PD-1 expression, reduced regulatory signatures, and acquisition of Th1-like features, contributing to persistent inflammation and tissue damage. Experimental models reveal a phase-dependent role for Tregs during infection. During the acute phase, transient contraction of the Treg compartment permits the development of protective effector responses required for parasite control. During chronic infection, restoration and maintenance of regulatory pathways, including IL-10, IL-33/ST2, CD39-mediated adenosine production, and G-CSF-associated mechanisms, limit immunopathology and promote tissue homeostasis. Figure created on Biorender.com

To formalize this synthesis, a vote counting analysis stratified by model type and infection phase was conducted (Supplementary Fig. 1). Across all studies amenable to directional classification (*n* = 23, excluding N/A and context-dependent), 17 (73.9%) reported a protective Treg role, a proportion significantly greater than chance (binomial test, *p* = 0.035). This predominance was driven particularly by chronic-phase studies, in which 8 of 9 studies (88.9%) reported protective Treg activity (*p* = 0.039). In contrast, acute-phase studies showed no significant directional predominance (4/7 protective, 57.1%; *p* = 1.000), consistent with the phase-dependent dual role described throughout this review (Fig. 4).

### Risk of bias assessment

Risk-of-bias assessment across all human studies was provided using the Cochrane RoB 2 tool, ROBINS-I, and the JBI Critical Appraisal Checklist (Supplementary Fig. 2). Panel A shows that the only randomized clinical trial (1/9; 11.1%) exhibited a predominantly high risk of bias, with major concerns in randomization and deviations from the intended intervention. Panel B indicates that the single non-randomized interventional study (1/9; 11.1%) presented an overall moderate risk, with unclear confounding control but low risk in outcome measurement and missing data. Most of the human evidence came from observational studies (7/9; 77.8%), evaluated with the JBI checklist (Panel C). These studies demonstrated consistently high methodological quality, with the majority of domains rated as “Yes” across inclusion criteria, measurement validity, confounder identification, and statistical analysis. Only a small subset of domains showed weaknesses, particularly in strategies to manage confounding (28.6% rated “No” or “Unclear”) and outcome assessment (14.3% rated “No”). Potential confounding factors represented an important source of uncertainty in the human evidence. Only two studies (Almeida et al., 2022; Damasio et al., 2019) explicitly reported procedures to exclude relevant co-infections or other immunomodulatory conditions that could influence Treg measurements. In the remaining studies, information regarding co-infections and comorbidities was incompletely reported or unavailable, which contributed to lower confidence in the interpretation of immune phenotypes and was considered during methodological quality assessment.

For experimental studies with mice, all studies scored between 6 and 7 points, resulting in a consistent classification of moderate methodological quality (Supplementary Fig. 3). Strengths included publication in peer-reviewed journals (Q1), clear presentation of results in the text (Q7), execution of methods described in sufficient detail (Q8), and general compliance with animal welfare guidelines (Q5). Common limitations were the absence of explicit randomization (Q2) and failure to report sample size calculations (Q4) or withdrawals/experimental attrition (Q9). Nonetheless, conclusions were generally aligned with reported data (Q10), and methodological constraints were consistent with typical immunoparasitology experimental designs. A detailed view of all risk-of-bias domains for every study is provided in Supplementary Figs. 2,3.

## Discussion

This systematic review synthesizes a complex and evolving body of evidence on the role of Tregs in *T. cruzi* infection. By integrating data from human clinical studies and experimental models, our analysis reveals that the impact of Tregs on pathogenesis is highly dynamic, dictated by the stage of infection, the specific tissue microenvironment, and the functional phenotype of the Treg subset involved. In human chronic Chagas disease (CD), a functional, stable Treg compartment is the hallmark of the asymptomatic (indeterminate) form, protecting the host from collateral tissue damage. On the other hand, experimental models elucidate the underlying mechanisms: during the acute phase, a physiological contraction of Treg activity is required to unleash the robust CD8⁺ T effector responses necessary for parasite control. Thus, disease outcome depends on the host’s capacity to transiently restrain Treg activity during acute infection and subsequently re-establish regulatory networks to preserve tissue integrity.

Evidence from human cohorts consistently delineates a dichotomy between the indeterminate (IND) and cardiac (CARD) clinical forms, driven mainly by the functional status of Tregs. Patients with the IND form typically exhibit a preserved immunoregulatory profile, characterized by Tregs with high expression of CD39, CTLA-4, and CD25, alongside robust IL-10 production [13, 22, 24]. This phenotype is directly correlated with preserved left ventricular function (LVEF) and reduced diastolic dysfunction. The CD39/adenosine pathway emerges as a critical molecular signature of this protective state; CD39⁺ Tregs effectively hydrolyze pro-inflammatory ATP into immunosuppressive adenosine, thereby limiting bystander tissue damage [6, 22]. In contrast, the progression to chronic cardiomyopathy is associated with a breakdown in this regulatory tolerance. Although an increase in the number of Treg cells has been observed in severe cardiac patients [14], these cells often display an “exhausted” or dysfunctional phenotype, marked by the upregulation of PD-1 and reduced suppressive capacity [22, 24]. Furthermore, genetic host factors may underpin these responses; specific polymorphisms in the FOXP3 promoter are associated with higher Treg expression and the protective IND phenotype, particularly in women, hinting at a sexually dimorphic regulatory potential [19].

Experimental models provide the temporal resolution needed to explain why regulatory suppression during *T. cruzi* infection must be dynamic. During the acute phase, effective parasite clearance requires a robust Th1 and cytotoxic CD8⁺ T response, which is antagonized by Treg activity. Consequently, successful acute control is marked by a physiological contraction of the Treg compartment; murine studies demonstrate that Tregs undergo limited expansion relative to effector cells and may be actively suppressed by the inflammatory environment [29, 30, 35]. However, this contraction must be precise: if it is too severe, or if surviving Tregs lose their lineage stability, lethal immunopathology ensues. Crucially, our review identifies the conversion of Tregs into a pathogenic ‘Th1-like’ phenotype (T-bet^+^, IFN-γ^+^) as a key driver of this failure [35]. We propose that this phenomenon represents the ‘Treg fragility’ described in recent tumor immunology literature, a state in which FOXP3⁺ Tregs persist but progressively lose suppressive capacity under sustained inflammatory stress, and may acquire partial effector functions [42]. Since in vivo Treg function is highly enhanced by specific antigen recognition [43, 44], characterizing the specific *T. cruzi* antigens that induce this contraction and phenotypic alteration could represent a substantial breakthrough. Uncovering these epitopes would not only clarify the mechanism of Treg instability but also pave the way for the development of novel immunotherapies and vaccines. Therefore, a favorable outcome of infection appears to be a stable population of Treg cells that allows for the activation of effector cells without causing uncontrolled inflammation.

This review identified a key set of Treg survival and stabilization factors, including granulocyte colony-stimulating factor (G-CSF), IL-2, and IL-33, which play a pivotal role in preserving the metabolic fitness of those cells, avoiding Treg fragility under *T. cruzi* inflammatory stress [25, 26, 35, 36, 38]. As infection transitions to the chronic phase, the role of Tregs shifts from permitting immunity to promoting tissue repair. Here, we highlight the critical role of tissue-repair Tregs (trTregs) and the IL-33/ST2 axis. In the skeletal muscle, trTregs are programmed for repair, producing amphiregulin and IL-10 in response to IL-33 [26, 45]. Disruption of this axis through ST2 deficiency leads to extensive tissue degeneration and fibrosis [25]. Similarly, IL-2 is indispensable for Treg maintenance through STAT5 signaling, sustaining FOXP3 transcription and metabolic fitness [46, 47]. Low-dose IL-2 therapy has been shown to expand functional Tregs and reduce myocarditis without compromising parasite control [36]. Furthermore, G-CSF, a hematopoietic growth factor, similarly expands and stabilizes Tregs by promoting STAT3-dependent survival and mobilization from the bone marrow [48, 49]. In *T. cruzi* infection, G-CSF acts as a stabilizer of the Treg lineage, promoting their survival and accumulation in the heart to mitigate fibrosis [38]. These findings suggest that chronic cardiomyopathy is also driven by a failure of these specific reparative mechanisms to heal the myocardium.

However, the mechanistic pathways discussed in this review, particularly those involving IL-2, IL-33/ST2, and G-CSF, are described qualitatively based on the experimental designs of the included studies. None of the studies reported standardized effect sizes, and dose-response relationships for these cytokine-based interventions were not consistently characterized across the literature. As a result, the relative contribution of each pathway to Treg stabilization or functional restoration cannot be formally ranked or compared. Future studies designed with dose-response paradigms and common outcome metrics would substantially strengthen the translational relevance of these mechanistic findings.

The association between Treg phenotype and clinical status has immediate translational implications. Notably, Tregs are being increasingly evaluated as biomarkers across multiple diseases. In autoimmune diseases such as type 1 diabetes and juvenile idiopathic arthritis, Treg gene signatures correlate with disease activity; in cancer, Treg abundance and phenotype predict therapeutic response; and in multiple myeloma, circulating Treg profiles identify high-risk patients [50–53]. These precedents highlight the potential of translating Treg-based monitoring into clinical practice. Specific Treg markers, particularly CD39, CD25^high^, and low PD-1, hold promise as biomarkers for monitoring disease progression and prognosis. Monitoring the ratio of functional (CD39⁺) to exhausted (PD-1⁺) Tregs could provide an immunological readout of cardiac risk and immunological status. Furthermore, therapeutic interventions can restore regulatory balance. Etiological treatment with benznidazole facilitates a shift in the Treg compartment from an activated, inflammatory state to a resting, homeostatic phenotype [21, 23]. Moreover, immunomodulatory strategies that reinforce natural regulatory pathways offer a promising adjunct to antiparasitic drugs. Candidates such as fenofibrate, pentoxifylline, and low-dose aspirin have been shown to modulate cytokine environments in ways that favor regulation [54–58]. More targeted approaches, such as low-dose IL-2 or IL-33 supplementation, could theoretically boost reparative Tregs to limit damage and fibrosis in patients with cardiac impairment.

Insights from other infections further contextualize these findings regarding the context-dependent role of Tregs. In *Leishmania major* infection, pathogen-specific Tregs sustain low-level parasite persistence, balancing protection and pathology [59]. In *Schistosoma mansoni*, Tregs expand alongside Tr1 cells to curb granulomatous reactions, with IL-10 and TGF-β as key mediators of fibrosis control [60]. In experimental cerebral malaria and cryptococcal meningoencephalitis, functional Tregs are essential to shield the brain from lethal, Th1-driven inflammatory damage [61, 62]. Conversely, in lethal *Toxoplasma gondii* infection, a decrease in Treg numbers and their acquisition of a pathogenic Th1-like phenotype is associated with fatal neuropathology [63]. In *T. cruzi* infection, CNS involvement is increasingly recognized. Human studies demonstrate that CD is associated with cognitive impairment independent of cardiac dysfunction [64]. Experimental models reveal brain parasitism, microglial activation, and neuroinflammation, but the regulatory mechanisms that control this response remain unexplored [65]. Given that *T. cruzi* induces thymic atrophy and alters lymphocyte migration [66], it is plausible that a failure of Treg surveillance in the brain contributes to the neurological sequelae of CD. Investigating the role of Tregs in controlling CNS inflammation in *T. cruzi* infection could unveil novel neuroprotective strategies [67].

This systematic review is, to our knowledge, the first to comprehensively synthesize the dual role of regulatory T cells in *T. cruzi* infection by integrating evidence from both human clinical studies and in vivo experimental models. However, the included evidence has significant limitations. The human studies are almost exclusively cross-sectional and restricted to peripheral blood, preventing definitive conclusions about causality or tissue-specific events. Several depletion studies utilized anti-CD25 antibodies, a method with the significant limitation of potentially depleting activated, CD25-expressing effector T cells in addition to Tregs. This high degree of heterogeneity precluded a formal statistical meta-analysis. Furthermore, the absence of harmonized outcome reporting and standardized quantitative measures prevented calculation of pooled effect estimates or evaluation of dose–response relationships across studies.

The experimental studies exhibit high heterogeneity in mouse strains (A/J, BALB/c, C57BL/6), sex, age (range of 4–12 weeks), parasite strains (Y, Tulahuén, Brazil, Colombiana), and inoculum, which undoubtedly contributes to conflicting reports. Differences in host genetic background likely contribute to part of the variability observed among experimental studies. C57BL/6 mice generally develop stronger type 1 inflammatory responses and more efficient parasite control, whereas BALB/c mice tend to display a more regulated immune profile [68]. A/J mice are highly susceptible to infection and frequently develop more severe disease manifestations [69]. These strain-specific immunological characteristics may influence Treg expansion, stability, tissue distribution, and suppressive function, complicating direct comparisons across experimental systems.

Importantly, the distinction between acute and chronic infection phases is not uniform across experimental models. The kinetics of parasite replication, tissue inflammation, and immune regulation vary according to parasite strain, inoculum size, route of infection, tissue analyzed, and host genetic background. Across the studies included in this review, the acute phase was generally evaluated between 7 and 30 days post-infection (dpi), whereas chronic infection was most frequently investigated between 60 and 150 dpi. However, these temporal boundaries were not consistently defined. For example, some studies reported persistent inflammatory responses and altered Treg dynamics beyond 30 dpi, while others observed partial restoration of regulatory pathways at earlier time points. Consequently, identical time points may represent distinct immunological states depending on the experimental system employed. This heterogeneity likely contributes to the variability observed in Treg frequency, phenotype, stability, and suppressive function across studies. Therefore, the temporal framework proposed in this review should be interpreted as a conceptual synthesis of recurrent patterns identified across multiple models, not as a universally applicable model of *T. cruzi* infection.

An additional source of heterogeneity may derive from unmeasured co-infections and comorbidities in human cohorts. Chronic helminth infections are particularly relevant in regions historically endemic for CD, as these parasites are potent inducers of regulatory immune networks characterized by expansion of FOXP3⁺ Tregs, increased IL-10 production, enhanced CTLA-4 expression, and suppression of type 1 inflammatory responses [70, 71]. Previous studies have demonstrated that helminth-driven immunoregulation can substantially modify T-cell phenotypes and influence the course of concurrent infectious diseases [72, 73]. Because most studies included in this review did not systematically screen participants for co-infections and co-morbidities, it remains possible that part of the variability observed in Treg frequency and function reflects differences in environmental exposures. Future clinical studies should incorporate systematic assessment of co-infections and major comorbidities to improve interpretation of immunoregulatory signatures in CD.

Collectively, experimental and human evidence depict *T. cruzi* infection as an example of temporal and spatial duality in Treg biology. During the acute phase, restrained Treg activity allows effector immunity to emerge, whereas during the chronic phase, functional regulatory networks become indispensable to contain inflammation and preserve tissular integrity. The timing, localization, and qualitative features of Tregs dictate tissue impairment and disease outcomes. Understanding the molecular and environmental cues that govern this balance opens new therapeutic avenues. Interventions targeting Treg survival and function, such as IL-2, IL-33 supplementation, or metabolic support of Tregs, may yield safer immunomodulation. Ultimately, the outcome of CD is determined by the host’s ability to orchestrate this delicate balance: to unleash effector responses sufficient for parasite control while preserving the regulatory circuits necessary for long-term tissue dynamics.

## Conclusions

This systematic review reveals that regulatory T cells (Tregs) exert a dual and temporally regulated function in *T. cruzi* infection. A transient reduction in Treg activity during the acute phase appears necessary to enable effective CD8⁺ T cell–mediated parasite control, whereas the subsequent restoration of a stable, suppressive, and tissue-repair oriented Treg pool is essential for long-term tolerance and prevention of inflammatory cardiomyopathy. Across human studies, specific Treg phenotypes, particularly CD39 expression and the activated-to-resting Treg ratio, emerge as refined indicators capable of distinguishing indeterminate from cardiac disease, emphasizing their promise as clinically relevant biomarkers. Experimental models further highlight key regulatory pathways, including IL-10, IL-2, G-CSF, and IL-33/ST2, as determinants of protection or pathology. Collectively, these findings support a conceptual framework in which Tregs act as dynamic modulators of disease tolerance, shaping the continuum from asymptomatic persistence to cardiomyopathy. Interventions that stabilize or reprogram these regulatory circuits may provide a foundation for precision immunotherapy in Chagas disease.

## Supplementary Information

Below is the link to the electronic supplementary material.

Supplementary Material 1.

## Data Availability

No datasets were generated or analysed during the current study.

## Declarations

### Protocol registration

The protocol for this systematic review was prospectively registered with PROSPERO (International Prospective Register of Systematic Reviews) under registration code: #CRD420251158158.
