# Supplementary material for: Dynamics and functional heterogeneity of FOXP3+ regulatory T cells in Trypanosoma cruzi infection: a systematic review
Source: Inflamm Res. 2026 Oct 12;75(1):234. doi: 10.1007/s00011-026-02330-6 (PMC13658323; doi:10.1007/s00011-026-02330-6)
Supplement: Supplementary file 1 [file 11_2026_2330_MOESM1_ESM.docx]

**Dynamics and Functional Heterogeneity of FOXP3+ Regulatory T Cells in *Trypanosoma cruzi* Infection: A Systematic Review**

Marcelo Eduardo Cardozo^1,2^, Tatyane Martins Cirilo^1^, Ana Laura Grossi de Oliveira^1,2^, Ana Rafaela Antunes-Porto^1^, Luisa Vitor do Braga Amaral^1^, José Bryan da Rocha Rihs^1^, Luisa Mourão Dias Magalhães^3,4^, Lilian Lacerda Bueno^1,2^, Fabiana Simão Machado ^2,5*^, Ricardo Toshio Fujiwara^1,2*^

^1^ Laboratory of Immunobiology and Parasite Control, Institute of Biological Sciences, Universidade Federal de Minas Gerais, Belo Horizonte, Brazil,

^2^ Post-graduation Program in Health Sciences: Infectious Diseases and Tropical Medicine, Faculdade de Medicina, Universidade Federal de Minas Gerais, Belo Horizonte, Brazil,

^3^ Laboratory of Interactions in ImmunoParasitology, Institute of Biological Sciences, Universidade Federal de Minas Gerais, Belo Horizonte, Brazil

^4^ Institute of Research in Mucosa and Skin (INCT Mucosa and Skin), Universidade Federal de Minas Gerais, Belo Horizonte, Brazil

^5^ Laboratory of Immunoregulation of Infectious Diseases, Institute of Biological Sciences, Universidade Federal de Minas Gerais, Belo Horizonte, Brazil

**Corresponding authors**

*Prof. Ricardo Toshio Fujiwara

Laboratory of Immunobiology and Parasite Control, Institute of Biological Sciences, Universidade Federal de Minas Gerais, Belo Horizonte, Brazil, 6627, Antônio Carlos Avenue, Pampulha Campus: Building E4/Room 173, Belo Horizonte, Brazil.

E-mail address: rtfujiwara@gmail.com (R.T. Fujiwara).

ORCID: https://orcid.org/0000-0002-4713-575X

*Prof. Fabiana Simão Machado

Laboratory of Immunoregulation of Infectious Diseases, Institute of Biological Sciences, Universidade Federal de Minas Gerais, Belo Horizonte, Brazil, 6627, Antônio Carlos Avenue, Pampulha Campus: Building O4/Room 190, Belo Horizonte, Brazil.

E-mail address: machadofs@gmail.com (F.S. Machado).

ORCID: https://orcid.org/0000-0001-9272-5209

**Supplementary information**

**Supplementary table 1**

**Search strategy in the different databases (PubMed, EMBASE, SCOPUS and LILACS)**

| **Search Strategy for PubMed** | **Search Strategy for EMBASE** | **Search Strategy for SCOPUS** | **Search Strategy for LILACS** |
| --- | --- | --- | --- |
| #1 "Chagas Disease"[Mesh] OR "American Trypanosomiasis"[Title/Abstract] OR "Trypanosoma cruzi"[Title/Abstract]  #2 "T-Lymphocytes, Regulatory"[Mesh]  OR "Regulatory T cell"[Title/Abstract]  OR "Regulatory T cells"[Title/Abstract]  OR "Regulatory T lymphocyte*"[Title/Abstract]  OR "Treg*"[Title/Abstract]  OR "Foxp3"[Title/Abstract]  OR "Forkhead box P3"[Title/Abstract]  OR "CD4+CD25+"[Title/Abstract]  #3 #1 AND #2 | ('chagas disease'/exp OR 'american trypanosomiasis':ab,ti OR 'trypanosoma cruzi':ab,ti)  AND  ('regulatory t lymphocyte'/exp OR 'regulatory t cell*':ab,ti OR 'regulatory t lymphocyte*':ab,ti OR 'treg*':ab,ti OR 'foxp3':ab,ti OR 'forkhead box p3':ab,ti OR 'cd4+cd25+':ab,ti) | (TITLE-ABS-KEY("Chagas Disease" OR "American Trypanosomiasis" OR "Trypanosoma cruzi"))  AND  (TITLE-ABS-KEY("Regulatory T cell*" OR "Regulatory T lymphocyte*" OR "Treg*" OR "Foxp3" OR "Forkhead box P3" OR "CD4+CD25+")) | (tw:("Doença de Chagas" OR "Enfermedad de Chagas" OR "Tripanossomíase Americana" OR "Tripanosomiasis Americana" OR "Trypanosoma cruzi"))  AND  (tw:("Linfócitos T Reguladores" OR "Linfocitos T Reguladores" OR "Células T Reguladoras" OR "Treg$" OR "Tregs$" OR "Foxp3" OR "CD4+CD25+")) |

**Supplementary Fig. 1. Vote counting analysis of the direction of Treg functional effect across 27 studies of *Trypanosoma cruzi* infection.** (A) Study-level classification. Each horizontal row represents one included study. Studies are ordered by model (human studies, n=9, above the dashed line; murine models, n=18, below) and, within each group, by assigned functional category. The filled circle on the right axis indicates the direction of the reported Treg effect for each study: protective (▶, teal), detrimental (◀, red), context-dependent (◆, amber), or not applicable (○, gray). "Protective" was assigned when Tregs were associated with reduced immunopathology, preserved cardiac function, or disease tolerance; "detrimental" when Tregs suppressed effector responses required for parasite control or were associated with worse outcomes; "context-dependent" when the direction of effect varied as a function of infection phase, treatment status, or host genetic background; and "N/A" when the study design did not permit directional assignment of Treg function. (B) Vote counting by study model. Horizontal stacked bars show the percentage of studies in each functional category, stratified by model type and overall. Studies classified as context-dependent or N/A were excluded from the binomial test. A one-sample binomial test was applied to assess whether the proportion of directionally classifiable studies reporting a protective Treg role differed significantly from 0.5 (H₀: P[protective] = 0.5). Overall (n=23 classifiable): 17 protective (73.9%), 6 detrimental (26.1%); p=0.035 (*); 95% CI [0.516, 0.898]. Human studies (n=8 classifiable): 7 protective (87.5%), 1 detrimental (12.5%); p=0.070 (ns); 95% CI [0.473, 0.997]. Murine models (n=15 classifiable): 10 protective (66.7%), 5 detrimental (33.3%); p=0.302 (ns); 95% CI [0.384, 0.882]. Significance: * p<0.05; ns = not significant. (C) Vote counting by infection phase. Same methodology as Panel B, with stratification by the primary infection phase evaluated in each study. "Both" indicates studies that evaluated both acute and chronic phases within the same experimental design. Acute phase (n=7 classifiable): 4 protective (57.1%), 3 detrimental (42.9%); p=1.000 (ns); 95% CI [0.184, 0.901]. Chronic phase (n=9 classifiable): 8 protective (88.9%), 1 detrimental (11.1%); p=0.039 (*); 95% CI [0.518, 0.997]. Both phases (n=7 classifiable): 5 protective (71.4%), 2 detrimental (28.6%); p=0.453 (ns); 95% CI [0.290, 0.963]. Accordingly, results should be cautiously interpreted in conjunction with the risk-of-bias assessment (Supplementary Figures 2–3). The non-significant binomial test in human studies reflects limited statistical power (n=8 classifiable). Similarly, the absence of directional predominance in acute-phase studies (p=1.000) is biologically consistent with the dual-role model proposed in this review, in which acute Treg restriction is physiologically necessary for parasite control.

**Supplementary Fig. 2. Risk of bias and methodological quality assessment of included human studies.** (A) Assessment of the randomized clinical trial (Vallejo et al., 2016) using the Cochrane Risk of Bias 2 (RoB 2) tool. The study was classified as having a high overall risk of bias, primarily driven by concerns regarding deviations from the intended intervention (D2) and missing outcome data (D3). (B) Assessment of the non-randomized interventional study (Llaguno et al., 2019) using the ROBINS-I tool. The study was judged as having a moderate overall risk of bias, with concerns noted in the domains of confounding (D1), participant selection (D2), and missing data (D5). (C) Critical appraisal of observational studies (cross-sectional and case-control) utilizing the Joanna Briggs Institute (JBI) Checklist. The heatmap displays the judgment for each domain across the seven included studies. Green indicates "Yes" (low risk), yellow indicates "Unclear", and red indicates "No" (high risk). While the majority of studies demonstrated robust methodology regarding inclusion criteria and outcome measurement, the identification of confounding factors (D5) and strategies to deal with confounders (D6) were the most consistent sources of potential bias.

**Supplementary Fig. 3. Risk of bias and methodological quality assessment of included animal studies.** The risk of bias was analyzed using the CAMARADES (Collaborative Approach to Meta-Analysis and Review of Animal Experimental Studies). Depicted results are the answers for all studies per question. Q1: Publication in a peer-reviewed journal and reporting; Q2: Random allocation to groups; Q3: Blinded assessment of outcome; Q4: Performing a sample size calculation; Q5: Compliance with animal welfare regulations; Q6: A statement of potential conflicts of interest; Q7: The results are presented and expressed in the text of the study, not only in figures, graphs, or tables; Q8: Execution of methods described in sufficient detail to permit its replication; Q9: Withdrawals from the study explained; Q10: The conclusion reflects the purpose of the research. Status: 1–2 points = weak, 3–4 points = medium, 5–7 points = moderate, and 8–10 points = strong.
